# TCR-Induced Tyrosine Phosphorylation at Tyr270 of SUMO Protease SENP1 by Lck Modulates SENP1 Enzyme Activity and Specificity

**DOI:** 10.3389/fcell.2021.789348

**Published:** 2022-02-02

**Authors:** Yun-Yi Li, Haohua Cen, Bei-Ni Gong, Siqi Mai, Qi-Long Wang, Sisi Mou, Yingqiu Li

**Affiliations:** Guangdong Province Key Laboratory of Pharmaceutical Functional Genes, MOE Key Laboratory of Gene Function and Regulation, State Key Laboratory of Biocontrol, School of Life Sciences, Sun Yat-sen University, Guangzhou, China

**Keywords:** TCR signaling, Lck kinase, tyrosine phosphorylation of SENP1, isopeptidase activity, endopeptidase specificity

## Abstract

Small ubiquitin-like modifier (SUMO) modification plays an important regulatory role in T cell receptor (TCR) signaling transduction. SUMO-specific proteases (SENPs) have dual-enzyme activities; they can both process SUMO precursors as endopeptidases and participate in SUMO deconjugation as isopeptidases. It remains unclear how the SUMO system, especially SENP1, is regulated by TCR signaling. Here, we show that Lck phosphorylates tyrosine 270 (Y270) of SENP1 upon TCR stimulation, indicating that SENP1 is a substrate of Lck. *In vitro* endopeptidase activity analysis showed that mutating SENP1 Y270 to either phenylalanine (F) to mimic the phosphorylation-defective state or to glutamate (E) to mimic the negative charge of tyrosine phosphorylation in the enzyme microenvironment did not change its endopeptidase activity towards pre-SUMO1. However, SENP1 Y270E but not Y270F mutation exhibited decreased endopeptidase activity towards pre-SUMO3. Through *in vivo* isopeptidase activity analysis by rescue expression of SENP1 and its Y270 mutants in a SENP1 CRISPR knockout T cell line, we found that SENP1 Y270F downregulated its isopeptidase activity towards both SUMO1 and SUMO2/3 conjugation by reducing SENP1 binding with sumoylated targets. While overexpression of SENP1 inhibited TCR-induced IL-2 production, overexpression of SENP1 Y270F enhanced it instead. In summary, TCR-induced Y270 phosphorylation of SENP1 may promote its isopeptidase activity and specifically decrease its endopeptidase activity against pre-SUMO3, which finely tunes activation of T cells.

## Introduction

Small ubiquitin-like modifier (SUMO) modification plays important roles in diverse cellular processes by regulation of most cellular signaling pathways. Abnormal sumoylation is related with human diseases, including cancer, neurodegenerative disorders, inflammation, and viral infection (Karhausen et al., 2021; Flotho and Melchior, 2013; Guerra et al., 2016; Liu and Shuai, 2009; Mendler et al., 2016; Seeler and Dejean, 2017). There are four mammalian SUMO paralogs: SUMO1, SUMO2, SUMO3, and SUMO4, consisting of 101, 95, 103, and 95 amino acids, respectively. SUMO2 and SUMO3 are nearly identical, only with differences of three amino acids in their mature forms (Johnson, 2004; Geiss-Friedlander and Melchior, 2007). SUMO1–3 covalently attach to particular lysine residues (often within a consensus site ψKxE/D, where ψ is an amino acid with a large hydrophobic side chain) of target proteins as post-translational modifiers (Creton and Jentsch, 2010), whereas the functional role of SUMO4 remains elusive. SUMOs are conjugated to target proteins by forming an isopeptide bond through a multistep enzymatic cascade, which involves a SUMO-activating enzyme E1 (a heterodimer composed of SAE1 and SAE2), a SUMO-conjugating enzyme E2 (Ubc9), and a SUMO E3 ligase (Chang and Yeh, 2020). Major differences among SUMOs comprise not only their expression levels but also their specificities to SUMO-specific proteases (SENPs) and to forming SUMO chains (Flotho and Melchior, 2013).

SUMOs are synthesized as inactive precursors and are cleaved by SENPs by removing several amino acids from the C-terminus of pre-SUMOs, with the exception of SUMO4, which contains Pro90 preventing its maturation by SENPs and then sumoylation (Owerbach et al., 2005). Sumoylation is reversible and highly transient due to the presence of SENPs. SENPs act as both endopeptidases and isopeptidases, and this dual function shows great significance during sumoylation cycle (Mikolajczyk et al., 2007). SENPs belong to the clan CE of cysteine proteases characterized by an adenain-like catalytic domain (Kim and Baek, 2009). It consists of SENP1–3 and SENP5–8 in SENP family, although SENP8 exhibits specificity for neddylation (another ubiquitin-like modification) instead of sumoylation (Nayak and Muller, 2014; Kunz et al., 2018).

SENPs share a highly evolutionary conserved catalytic domain in C-terminus, while their variable N-terminal regions result in distinct spatial distributions and even substrate specificities (Xu et al., 2006). SENPs specifically distinguish precursors or conjugates of distinct SUMO paralogs. Hydrolysis of SUMO1–3 precursors by SENP1 can be detected *in vitro*. Preferring pre-SUMO1 > pre-SUMO2 > pre-SUMO3, SENP1 hydrolyzes SUMO1 precursor in a more efficient way (Shen et al., 2006), whereas SENP2 displays a slight preference for SUMO2 precursor (Reverter and Lima, 2004; Reverter and Lima, 2006). Besides, SENP1 is indispensable for desumoylation of SUMO1-modified but not of SUMO2/3-modified proteins (Sharma et al., 2013). SENP3 and SENP5 preferentially remove SUMO2/3 conjugates from protein substrates (Gong and Yeh, 2006; Hickey et al., 2012). SENP6 and SENP7 are proficient in deconjugating di-SUMO2/3 and poly-SUMO2/3 chains (Lima and Reverter, 2008; Shen et al., 2009; Alegre and Reverter, 2011).

Sumoylation is important in regulation of T cell receptor (TCR) signaling (Ding et al., 2016; He et al., 2021; Liu et al., 2015; Wang et al., 2015, 2019,; Xiong et al., 2019). However, how the SUMO system is regulated by TCR is unclear. Here, we have demonstrated that upon TCR stimulation, Lck phosphorylates tyrosine 270 of SENP1 in its N-terminus, the non-catalytic domain, which in turn fine-tunes the enzyme activity and specificity of SENP1 and ensures appropriate activation of T cells. Therefore, we have revealed that TCR induces phosphorylation of SENP1 and then modifies isopeptidase activity and substrate specificity of SENP1, providing a novel mechanism for meticulous regulation of TCR signaling.

## Materials and Methods

### Plasmids

The cDNAs encoding SENP1 (NP_001254524.1), Lck (NP_001036236.1), ZAP70 (NP_001365523.1), SUMO1 (NP_003343.1), and SUMO3 (NP_008867.2) were amplified by PCR from Jurkat E6.1 T cell cDNA and cloned into the vectors pcDNA3.1-c-Myc (Invitrogen), YFP-C1 (BD), and pGEX-4T-2 (Sigma-Aldrich), respectively. Specific point mutations of SENP1 were introduced by site-directed mutagenesis with a QuikChange Site-Directed Mutagenesis Kit (Stratagene). Primers are shown in Supplementary Table S1.

### Antibodies and Regents

Antibodies to SENP1 (C-12), Lck (3A5), RanGAP1 (C-5), c-Myc (9E10), β-actin (C4), and GAPDH (FL-335) were from Santa Cruz Biotechnology. Antibodies to SENP1 (ab108981), SUMO1 (ab32058), and SUMO2/3 (ab3742) were from Abcam. Antibodies to HA (C29F4) and glutathione S-transferase (GST) were from Cell Signaling Technology. M2 antibody to Flag (F1804) was from Sigma-Aldrich, and 4G10 (05-1050) was from Millipore. Anti-human CD3 (OKT3) and anti-human CD28 (CD28.2) were from eBioscience. Goat anti-mouse IgG (31160) was from Thermo Fisher Scientific. Horseradish peroxidase-conjugated secondary antibodies were from Jackson ImmunoResearch.

### Cell Culture, Transfection, and Stimulation

Human leukemia Jurkat T cell line E6.1 cells, TAg cells, JCaM1.6 cells, and *SENP1* ^*−/−*^ cells were cultured in RPMI 1640 medium (HyClone Logan, UT, United States) supplemented with 10% (vol/vol) fetal bovine serum (FBS, Gibco) and 100 U/ml each of penicillin and streptomycin (Life Technologies) at 37°C and 5% CO_2_. Cells in a logarithmic growth phase were transfected by nucleofection (Lonza 4D Nucleofector™ system). After transfection, cells were cultured for 48 h before harvest. HEK293T cells were cultured in DMEM (HyClone) containing penicillin, streptomycin, and 10% FBS. Transfections were carried out with PEI (Sigma-Aldrich). For TCR stimulation with antibodies, cells were washed with serum-free RPMI 1640 medium and stimulated with 5 μg/ml anti-CD3 and 2 μg/ml anti-CD28, which were crosslinked with goat anti-mouse IgG (5 μg/ml). For pervanadate treatment, cells were incubated with tyrosine phosphatase inhibitor pervanadate (10 mM) for 20 min before harvest.

### Immunoprecipitation and Immunoblotting

After washing with ice-cold PBS, cells were lysed in lysis buffer (1% Nonidet P40, 150 mM NaCl, 20 mM Tris-HCl, pH 7.5, and 5 mM EDTA), supplemented with protease inhibitors (10 μg/ml aprotinin, 10 μg/ml leupeptin, and 1 mM PMSF) and phosphatase inhibitors (5 mM sodium pyrophosphate and 1 mM Na_3_VO_4_). After clearance by centrifugation, whole-cell lysates were incubated overnight with 0.6 µg of antibodies, and proteins were immunoprecipitated for an additional 4 h at 4°C with protein G Sepharose beads (GE healthcare) with gentle shaking. Immunoprecipitates were extensively washed five times with lysis buffer and were separated by SDS-PAGE. The immunoprecipitated proteins were transferred onto a PVDF membrane and probed overnight at 4°C with primary antibodies, followed by incubation for 1 h at RT with HRP-conjugated secondary antibodies. Signals were visualized by enhanced chemiluminescence (ECL; GE Healthcare) and were exposed to X-ray film or on the ChemiDoc XRS+ system (Bio-Rad). Densitometry analysis was performed with ImageJ software.

### Mass Spectrometry

After immunoprecipitation with SENP1 antibody in Jurkat E6.1 T cells, the immunoprecipitates were resolved by SDS-PAGE. Gel bands of interest were excised and subjected to tryptic digestion. After the desalting procedure, the peptides were analyzed by liquid chromatography-tandem MS. The peptides were analyzed by the LTQ Orbitrap Elite system and EASY-nLC 1000 system (Thermo Fisher Scientific), and then proteins in SENP1 immunoprecipitates were identified with the Protein Discovery software. Mass spectrometry proteomic data from this study are deposited to the ProteomeXchange-PRIDE repository (https://www.ebi.ac.uk/pride/archive/) and assigned the dataset number PXD029720.

### CRISPR/Cas9 Gene Editing

LentiCRISPRv2 (one vector system) coexpressing a mammalian codon-optimized Cas9 nuclease along with sgRNA to facilitate editing a SENP1 genomic fragment was transfected in the Jurkat E6.1 T cell line (Shalem et al., 2014; Sanjana et al., 2014). Then, 48 h after transfection, green fluorescent protein (GFP)-positive cells were sorted on a flow cytometer (BD FACSAria II) and seeded individually into 96-well plates. After extended culture, candidate monoclonal cell lines were selected by screening using western blot. The identifications of SENP1 genome-edited cell line are shown in Figure 5 and Supplementary Figure S3.

### GST Pull-Down Assay

GST fusion proteins were expressed in BL21 *E. coli* cells after induction with IPTG (Sangon Biotech). Bacteria were resuspended in lysis buffer (1× PBS pH 7.4, protease inhibitors, and 1% Triton X-100). Bacterial extracts were sonicated for 10 min and centrifuged. GST fusion proteins were purified from bacterial lysates by incubating with glutathione Sepharose beads (GE Healthcare). The precipitates were washed three times with lysis buffer, then incubated at 4°C overnight with cell lysates from HEK293T cells. Coomassie Brilliant Blue staining was used as loading control.

### *In Vitro* Endopeptidase Activity Assay

*In vitro* endopeptidase activity assay was achieved as previously described (Madu et al., 2013; Xu and Au, 2005). Briefly, GST-SUMO-GFP (Gs-S-Gf) and SENP1 proteins [wild type (WT) or mutants] were expressed in *E. coli* BL21 cells and purified. To assay the hydrolysis activity, eluted Gs-S-Gf proteins were incubated in the absence (control) or presence of SENP1 WT or its mutants (immobilized on glutathione Sepharose beads), respectively, and then incubated in assay buffer (150 mM NaCl, 10 mM Tris-HCI, and 10 mM DTT) at 37°C for 20 min and boiled in SDS loading buffer, followed by SDS-PAGE and Coomassie staining to analyze.

### *In Vitro* Isopeptidase Activity Assay

*SENP1* ^*−/−*^ T cells generated by CRISPR/Cas9 system were lysed in lysis buffer supplemented with 20 mM N-ethylmaleimide (Sigma-Aldrich). After clearance by centrifugation, 1% SDS (vol/vol) was added to the supernatants. Proteins were dissociated by incubation at 90°C for 10 min, followed by dilution (1:10) with ice-cold lysis buffer immediately. Endogenous total RanGAP1 (including RanGAP1 and RanGAP1-SUMO1) was immunoprecipitated from the above lysis buffer at 4°C overnight with anti-RanGAP1 antibody and immobilized on protein G Sepharose beads. After extensive washing with lysis buffer, immunoprecipitated proteins were equally divided into aliquots as reaction substrate, followed by incubation with GST-SENP1 WT or its mutants (immobilized on glutathione Sepharose beads) in reaction buffer (150 mM NaCl, 10 mM Tris-HCI, and 2 mM DTT) at 37°C for 20 min, respectively. The reaction was stopped by adding SDS loading buffer, followed by SDS-PAGE and immunoblotting to analyze.

### 3D Structure Modeling

The 3D structure of SENP1 (full length, 1–644 aa) was predicted using I-TASSER (https://zhanglab.ccmb.med.umich.edu/I-TASSER/) (Roy et al., 2010; Yang et al., 2015, Yang and Zhang, 2015). The predicted structures of SENP1 Y270F and Y270E mutation were acquired using Missense 3D (http://missense3d.bc.ic.ac.uk/missense3d/) (Ittisoponpisan et al., 2019). The display of structures and measurement of distances were performed by PyMOL software.

### Flow Cytometry

To examine the expression level of SENP1, Jurkat E6.1 and *SENP1* ^*−/−*^ T cells were collected, washed with 1% FBS in PBS buffer, and then permeabilized (Cytofix/Cytoperm Plus; BD) at 4°C for 20 min, followed by washing with 1% FBS/0.5% saponin in PBS buffer. After being sequentially stained with anti-SENP1 antibody on ice overnight and with cross-adsorbed secondary antibody (Alexa Fluor 647) at 4°C for 1 h, the cells were washed three times and resuspended in 1% FBS in PBS buffer, and then examined by using the CytoFLEX flow cytometer (Beckman).

### Real-Time PCR

Total RNA was extracted from Jurkat E6.1 and *SENP1* ^*−/−*^ T cells using TRIzol reagent (Magen) according to the manufacturer’s protocol. RNA was eluted with RNase-free water and quantified using a NanoDrop 2000 Spectrophotometer (Thermo Fisher Scientific). First-strand cDNA was synthesized by reverse transcription with Hifair III 1st Strand cDNA Synthesis Kit (gDNA digester plus, YEASEN, CHINA). Real-time PCR was run on a LightCycler 480 system (Roche) with Hieff qPCR SYBR Green Master Mix (YEASEN, CHINA) in triplicate. Real-time PCR primers sequences are shown in Supplementary Table S1. The relative quantity of mRNA was normalized with GADPH, using the comparative 2^–ΔΔCt^ method.

### Enzyme-Linked Immunosorbent Assay

Jurkat E6.1 T cells transfected with empty vector or YFP-SENP1 WT, Y270F, Y270E, and C603S were stimulated for 24 h with anti-CD3 plus anti-CD28, and the determination of the concentration of IL-2 in cultured supernatants by ELISA was achieved as described (Wang et al., 2019,^2015^). Briefly, a 96-well plate was coated with capture antibody to IL-2 at 4°C overnight. After pre-blocking at RT for 1 h, triplicates of standards and samples were then incubated at RT for 2 h in the plate. Incubation with detection antibody to IL-2 at RT for 1 h was done, followed by incubation with avidin-HRP at RT for 30 min. The amount of bound avidin was then assessed with TMB peroxidase that was acidified by 2N H_2_SO_4_. The absorbance of each well at 450 and 570 nm was then measured with a spectrophotometric plate reader (BioTek).

### Statistical Analysis

Data is representative of three independent experiments. GraphPad Prism 5.0 software was used for graphs and statistical analysis, performed with a two-tailed, unpaired Student’s *t*-test. For all tests, statistical significance was quantified by *p*-values. When *p*-values were below 0.05 (*), 0.01 (**), 0.001 (***), or 0.0001 (****), differences were considered statistically significant. In the figures, *p*-values are indicated as follows: *p < 0.05; **p < 0.01; ***p < 0.001; ****p < 0.0001, and ns, not significant. Graphs represent mean ± standard error of the mean (SEM).

## Results

### Lck Associates With SENP1

**FIGURE 1:**
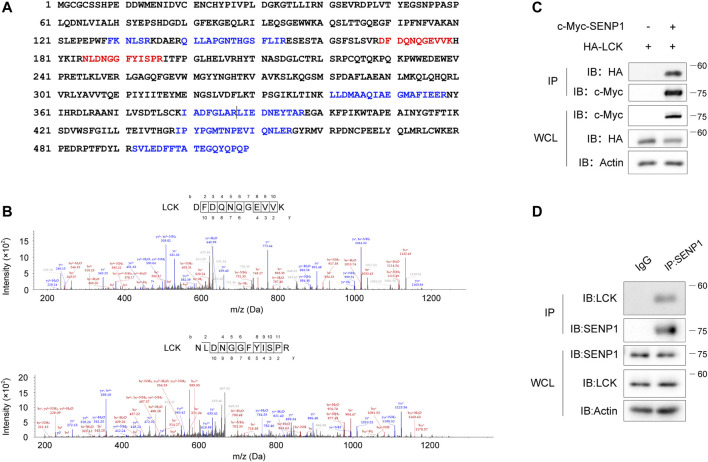
Lck associates with SENP1. **(A)** Mapping of nine Lck peptides (highlighted in blue or red) on Lck full-length protein sequence. The nine Lck peptides were identified by mass spectrometry analysis of SENP1-immunoprecipitated complex from Jurkat E6.1 T cells (two consecutive peptides were separated by a vertical line). Identified Lck peptides with higher frequencies were marked red. **(B)** Fragmentation spectrum of the two Lck peptides with higher frequencies in **(A)**. *m*/*z*, mass/charge ratio. **(C)** Coimmunoprecipitation of exogenously expressed c-Myc-SENP1 and HA-Lck (top) and immunoblots of whole-cell lysates (WCL, bottom) of HEK293T transfectants. IP, immunoprecipitation. **(D)** Coimmunoprecipitation of endogenous SENP1 and Lck in Jurkat E6.1 T cells (top) and immunoblots of Jurkat E6.1 T cell lysates (WCL, bottom).

To investigate whether SENP1 is regulated by TCR signaling, we first analyzed SENP1-associated proteins in T cells. In mass spectrometric analysis, nine peptides of Lck were shown in SENP1-immunoprecipitated complex from Jurkat E6.1 T cells (Figure 1A), and two of them had higher frequencies (Figure 1B), suggesting Lck may interact with SENP1. Coimmunoprecipitation analysis verified the association between overexpressed SENP1 and Lck in HEK293T cells (Figure 1C) and between endogenous SENP1 and Lck in Jurkat E6.1 T cells (Figure 1D).

### TCR Induces Phosphorylation of SENP1 by Lck

**FIGURE 2:**
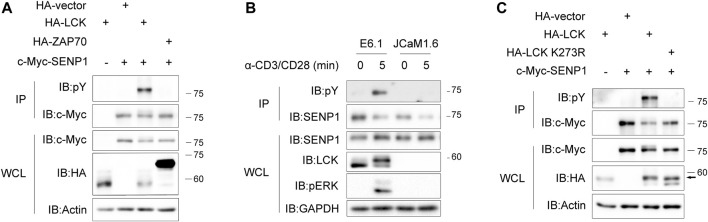
T cell receptor (TCR) induces phosphorylation of SENP1 by Lck. **(A)** Anti-phospho-tyrosine (pY) immunoblots of c-Myc-SENP1 immunoprecipitates from HEK293T cells cotransfected with c-Myc-SENP1 and HA-Lck or HA-ZAP70 (top) and immunoblots of WCL of HEK293T transfectants (bottom). **(B)** Anti-pY immunoblots of endogenous SENP1 immunoprecipitates from Jurkat E6.1 or Lck-deficient JCaM1.6 cells costimulated with anti-CD3 plus anti-CD28 for the indicated times (top) and immunoblots of WCL of Jurkat E6.1 or Lck-deficient JCaM1.6 cell lysates (bottom). **(C)** Analysis of Lck kinase activity for tyrosine phosphorylation on SENP1. Anti-pY immunoblots of c-Myc-SENP1 immunoprecipitates from HEK293T cells cotransfected with c-Myc-SENP1 and HA-Lck or HA-Lck K273R (top) and immunoblots of WCL of HEK293T transfectants (bottom).

Since Lck is a crucial tyrosine kinase in TCR proximal signaling (Shah et al., 2016), we next tested if SENP1 could be phosphorylated by Lck as a substrate. In HEK293T cells, tyrosine phosphorylation of exogenous SENP1 was detected when SENP1 was coexpressed with Lck, whereas it was undetectable when SENP1 was coexpressed with ZAP70, an important downstream kinase of Lck (Figure 2A). In Jurkat E6.1 T cells, anti-CD3 plus anti-CD28 costimulation induced tyrosine phosphorylation of endogenous SENP1; however, this induced phosphorylation disappeared in Lck-deficient JCaM1.6 cells (Figure 2B). We further cotransfected SENP1 with Lck or its kinase dead mutant (K273R) in HEK293T cells and observed that SENP1 was tyrosine phosphorylated only when cotransfected with Lck WT but not with its K273R mutant (Figure 2C), indicating that the kinase activity of Lck was essential for SENP1 phosphorylation. Together, these results demonstrated that SENP1 could be phosphorylated by tyrosine kinase Lck but not ZAP70.

### Tyr270 on SENP1 Is the Key Phosphorylation Site by Lck

**FIGURE 3:**
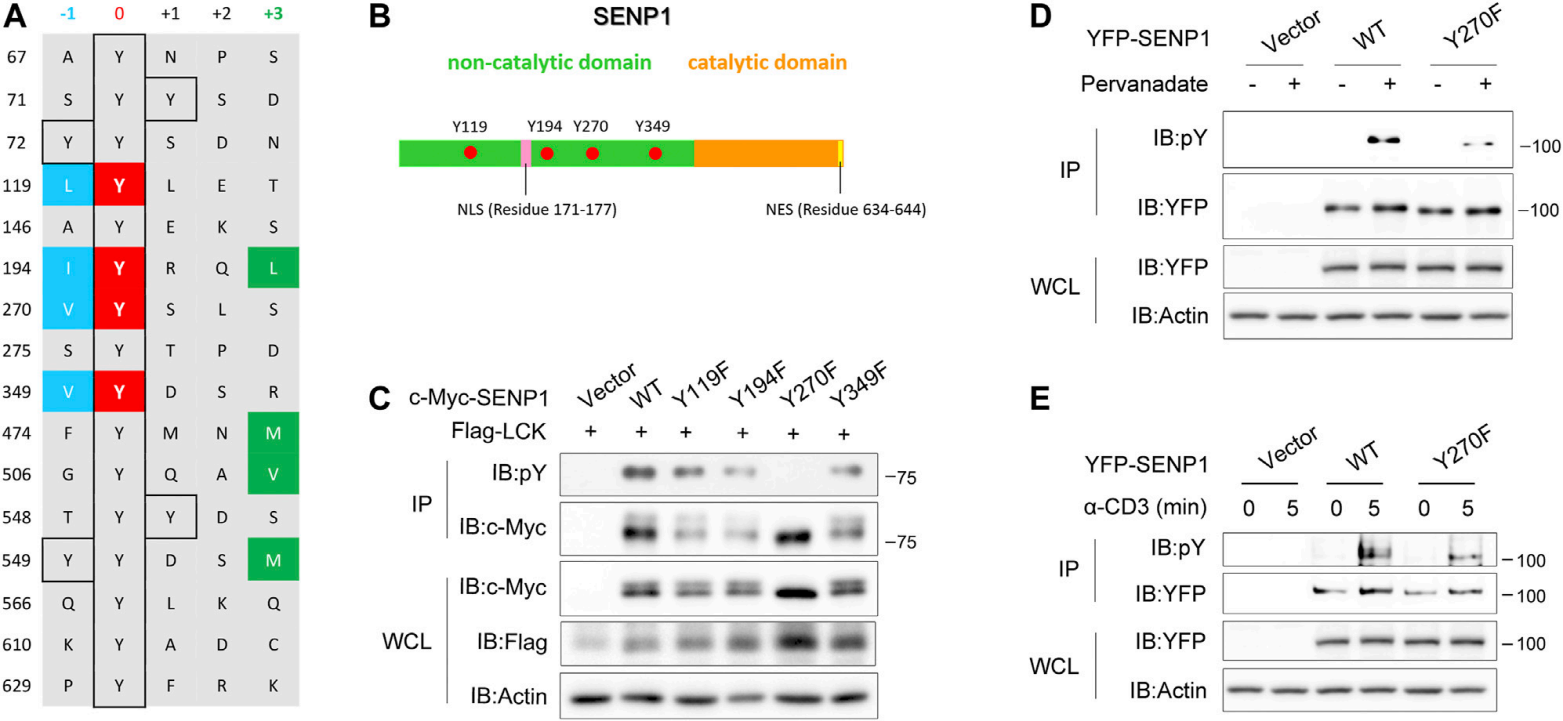
Tyr270 on SENP1 is the key phosphorylation site by Lck. **(A)** Prediction of potential phosphorylation sites on SENP1 according to the sequence preference of Lck. Y119, Y194, Y270, and Y349 shown in red are candidate phosphorylation sites on SENP1. **(B)** Schematic illustration of SENP1 with the locations of Y119, Y194, Y270, and Y349. NLS, Nuclear localization sequence; NES, nuclear export sequence. **(C)** Anti-pY immunoblots of anti-c-Myc immunoprecipitates from HEK293T cells cotransfected with Flag-Lck and c-Myc-SENP1 WT or its mutants Y119F, Y194F, Y270F, and Y349F, respectively (top), and immunoblots of WCL of HEK293T transfectants (bottom). **(D)** Immunoblot analysis of pervanadate (tyrosine phosphatase inhibitor)-induced tyrosine phosphorylation on YFP-SENP1 in Jurkat TAg cells. Anti-pY immunoblots of YFP-SENP1 immunoprecipitates from Jurkat TAg cells transfected with YFP-SENP1 WT or Y270F mutant followed by treatment with pervanadate at 37°C for 20 min (top), and WCL were immunoblotted as indicated (bottom). **(E)** Immunoblot analysis of anti-CD3-induced tyrosine phosphorylation on YFP-SENP1 in Jurkat TAg cells. Anti-pY immunoblots of YFP-SENP1 immunoprecipitates from Jurkat TAg cells transfected with YFP-SENP1 WT or Y270F mutant followed by stimulation with anti-CD3 for the indicated times (top), and WCL were immunoblotted as indicated (bottom).

Regarding phosphorylation sites, Lck has a strong preference for Ile, Leu, or Val at the −1 position, whereas it cannot tolerate Asp at this position, and Lck has a moderate preference for bulky hydrophobic residues at the +3 position (Shah et al., 2018; Shah et al., 2016). To identify the crucial phosphorylation site on SENP1 by Lck, we checked all tyrosine residues and their −1 and +3 position residues on SENP1 and noticed that four tyrosine residues Tyr119, Tyr194, Tyr270, and Tyr349 are in accordance with the rule of Lck phosphosite preference (Figure 3A). Interestingly, all of them are located in the N-terminal non-catalytic domain of SENP1 (Figure 3B). By mutating tyrosine to phenylalanine, we constructed Y119F, Y194F, Y270F, and Y349F mutants of SENP1, respectively. Through cotransfection of SENP1 YF mutants separately with Lck in HEK293T cells, we examined the effect of each YF mutation on phosphorylation of SENP1 by Lck. We found that Lck-mediated tyrosine phosphorylation of SENP1 remarkably disappeared when mutating Tyr270 to Phe (Figure 3C). Similarly, Y270F mutation notably decreased tyrosine phosphorylation of SENP1 induced by pervanadate (tyrosine phosphatase inhibitor) treatment in Jurkat TAg cells (Figure 3D). However, TCR-induced phosphorylation of SENP1 only reduced modestly with anti-CD3 stimulation (Figure 3E), suggesting that other tyrosine sites of SENP1 may also be phosphorylated upon TCR stimulation. SENP1 Tyr270 was a middle conserved site among various species, as its conservative index is 0.7 in alignment of 58 species in the database of VarSite; meanwhile, SENP1 Tyr270 is completely conserved in primates (Supplementary Figure S1), suggesting Y270 phosphorylation might be related to primate-specific immune responses.

### SENP1 Y270E Specifically Decreases Its Endopeptidase Activity Towards Pre-SUMO3 *In Vitro*

**FIGURE 4:**
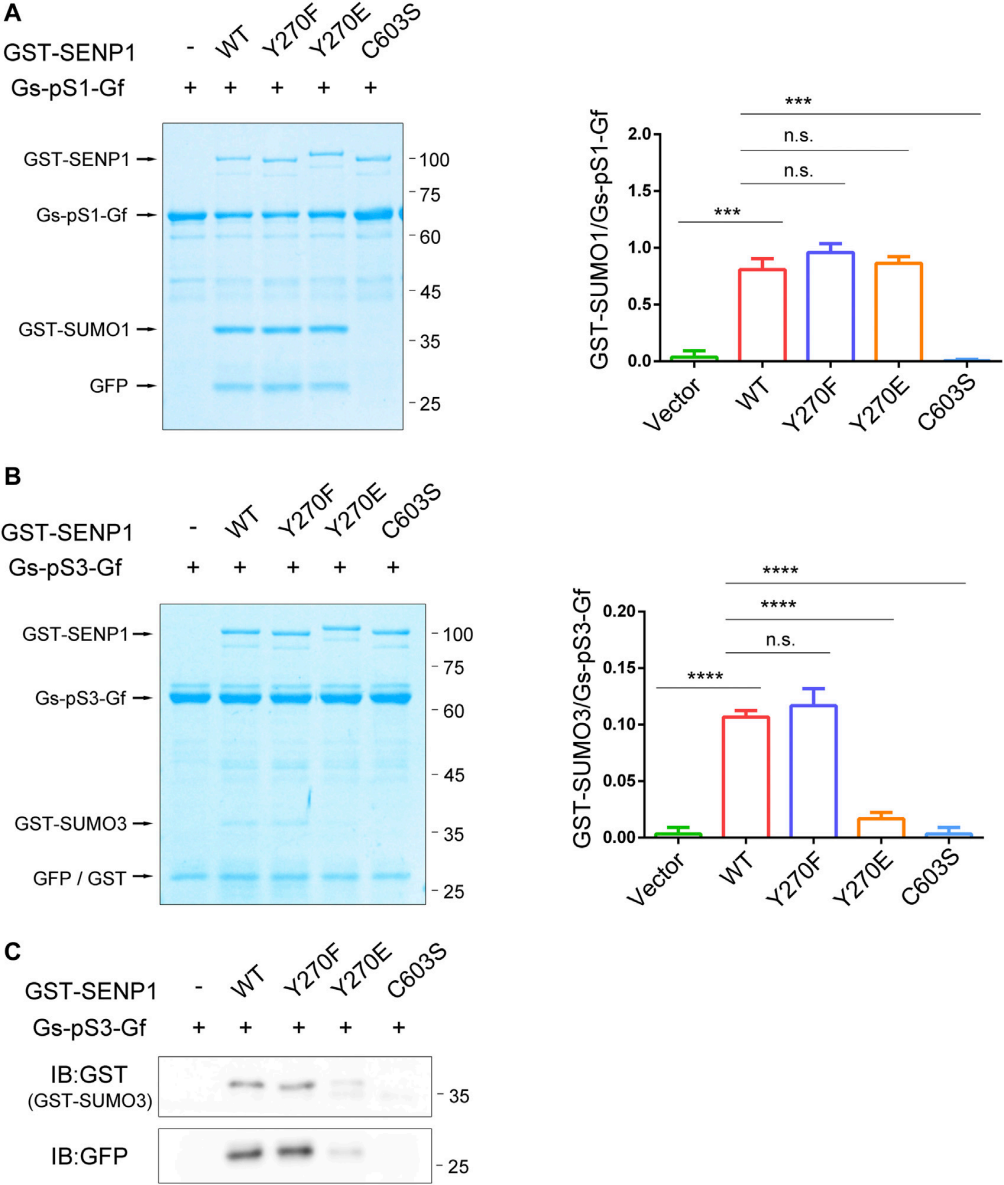
SENP1 Y270E specifically decreases its endopeptidase activity towards pre-SUMO3 *in vitro*. **(A,B)** *In vitro* SENP1 endopeptidase activity assay with purified glutathione S-transferase (GST)-preSUMO1-GFP (Gs-pS1-Gf, **(A)** or GST-preSUMO3-GFP proteins (Gs-pS3-Gf, **(B)** as substrates. GST-SENP1 WT and its Y270F, Y270E, and C603S mutants were incubated with Gs-pS1-Gf or Gs-pS3-Gf at 37°C for 20 min, respectively, followed by SDS-PAGE and Coomassie staining to analyze the reaction products processed by different forms of SENP1. The densitometric quantification of the ratios of GST-SUMO/Gs-pS-Gf (product/substrate) is shown on the right. n.s., not significant, *p < 0.05, **p < 0.01, ***p < 0.001, ****p < 0.0001 (two-tailed unpaired Student’s *t*-test). **(C)** Samples in **(B)** were also immunoblotted with anti-GST (GST-SUMO3, top) and anti-GFP (bottom) antibody.

Without characteristic features, the N-terminal regions of SENPs presumably control their enzymatic properties intrinsically (Mikolajczyk et al., 2007). We questioned if SENP1 Y270 phosphorylation could regulate its enzyme activity. Firstly, we assessed the impact of SENP1 Y270 phosphorylation on its endopeptidase activity by an *in vitro* assay (Figures 4A,B). We fused full-length SUMO1, i.e., precursor with GST at its N-terminus, with GFP at C-terminus to construct the recombinant SUMO1 precursor simultaneously fused with two tags (GST-preSUMO1-GFP or Gs-pS1-Gf, 64 kDa) and used it as a substrate of SENP1 endopeptidase according to the literatures (Xu and Au, 2005; Madu and Chen, 2012). Proteolytic cleavage of Gs-pS1-Gf by SENP1 yields two products, GST-SUMO1 (GST fused with mature SUMO1, approximately 38 kDa) and GFP moieties (HSTV peptide fused to N-terminus of GFP, about 26 kDa). The ratio of product/substrate reflects the reaction efficiency (Xu and Au, 2005; Madu and Chen, 2012). As expected, the capability of SENP1 to remove C-terminal GFP moiety from Gs-pS1-Gf was abolished by the catalytic inactive C603S mutation. However, neither a tyrosine phosphorylation-deficient Y270F mutant nor a Y270E mutant (which mimics the negative charge of tyrosine phosphorylation in the enzyme microenvironment) of SENP1 had any impact on its endopeptidase activity towards pre-SUMO1, indicating that Y270 phosphorylation on SENP1 did not regulate its endopeptidase activity towards pre-SUMO1.

Since SENP1 prefers pre-SUMO1 > pre-SUMO2 > pre-SUMO3, we guessed that SENP1 Y270 phosphorylation might also not affect its endopeptidase activity towards pre-SUMO2 or pre-SUMO3. We further performed an *in vitro* assay against SUMO3 precursor Gs-pS3-Gf (Xu and Au, 2005; Mikolajczyk et al., 2007). Due to the substrate preference of SENP1 endopeptidase activity, the bands related to GST-SUMO3 were faint. Unlike Gs-pS1-Gf, there was a band near above 25 kDa in all lanes when using Gs-pS3-Gf even in the absence of GST-SENP1 (labelled with GFP/GST in Figure 4B). Western blot showed that the Gs-pS3-Gf protein could be cleaved to generate GST-tag protein for uncertain reason (Supplementary Figure S2); thus, the band near above 25 kDa contained mixed proteins of GFP moiety (VPESSLAGHSF peptide fused to the N-terminus of GFP) and GST-tag. Nevertheless, we performed endopeptidase activity assay towards pre-SUMO3 with Coomassie staining and immunoblotting at the same time (Figures 4B,C). As expected, GST-SUMO3 could not be detected when reacting with SENP1 C603S mutant. Interestingly, GST-SUMO3 could not also be detected when reacting with SENP1 Y270E mutant, suggesting that SENP1 Y270E specifically decreases its hydrolytic ability to SUMO3 precursor.

The above data indicates that tyrosine phosphorylation on Y270 of SENP1 may modulate its endopeptidase activity with selectivity, which has no effects on its endopeptidase capacity toward pre-SUMO1, but decreases its endopeptidase activity against pre-SUMO3.

### SENP1 Y270 Phosphorylation Influences Its Isopeptidase Activity Both *In Vivo* and *In Vitro*

**FIGURE 5:**
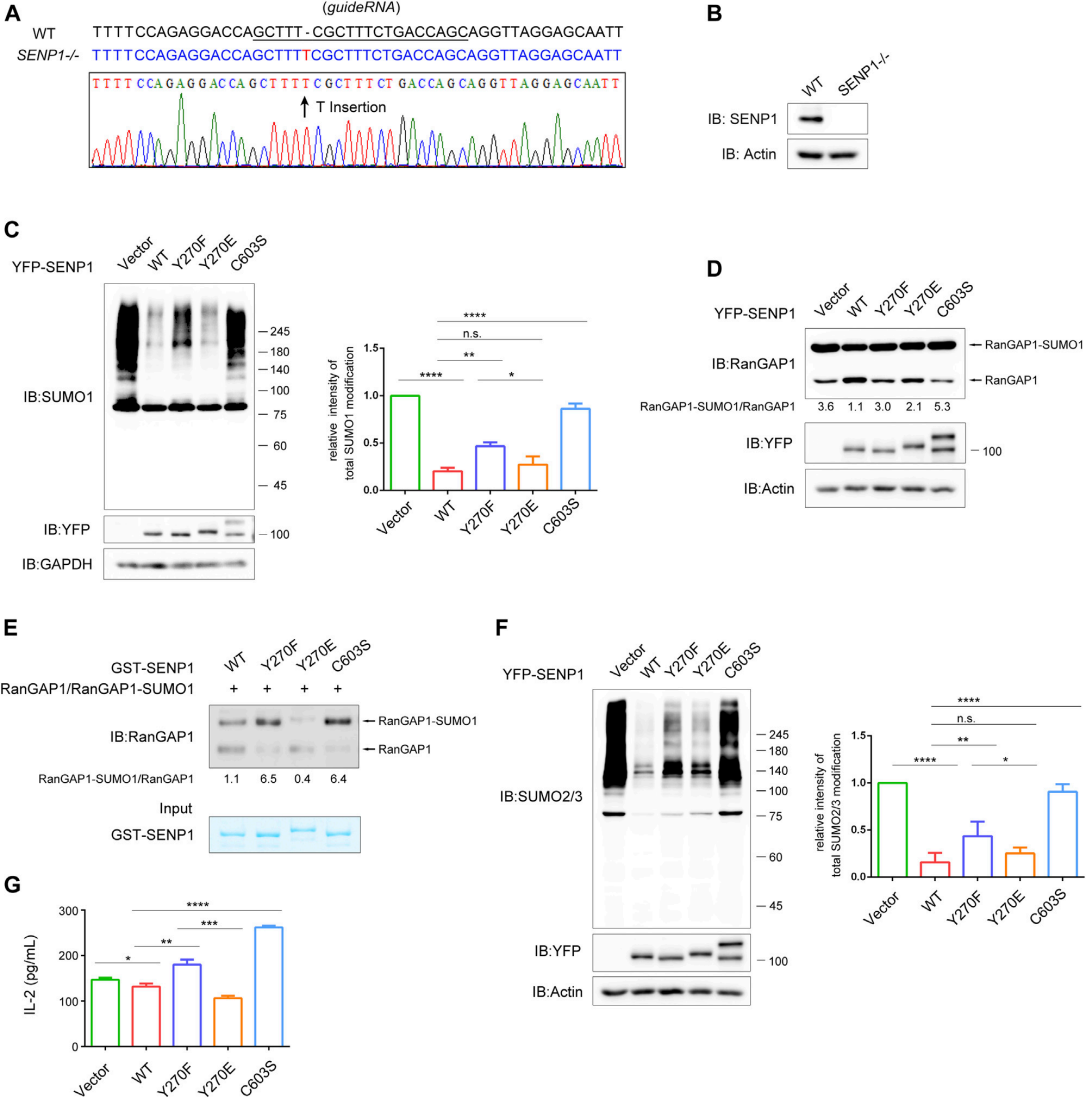
SENP1 Y270 phosphorylation influences its isopeptidase activity both *in vivo* and *in vitro*. **(A)** Sequence alignment of SENP1 genomic fragment (WT) and the CRISPR/Cas9-edited SENP1 genomic fragment in the *SENP1* ^*−/−*^ T cells (top). The underlined sequence represents the sgRNA. **(B)** The expression levels of SENP1 in WT and *SENP1* ^*−/−*^ T cells examined by western blot. **(C)** *In vivo* SUMO1-profiling assay of *SENP1* ^*−/−*^ T cells with rescue expression of YFP-SENP1 WT or its mutants or empty vector. GAPDH was blotted as loading control. The densitometric quantification of total SUMO1 modification (the relative intensity of the control was set as 1) is shown on the right. **(D)** *In vivo* isopeptidase activity analysis of SENP1 WT and its mutants. With rescue expression in *SENP1* ^*−/−*^ T cells as in **(C)**, cell lysates were analyzed for endogenous total RanGAP1 (including RanGAP1 and RanGAP1-SUMO1) by western blot (top). The densitometric quantification of the ratio of RanGAP1-SUMO1/RanGAP1 is shown below. The expression of SENP1 WT and its mutants and actin (loading control) are shown (bottom). **(E)** *In vitro* isopeptidase activity assay of SENP1 WT and its mutants. The experiment was performed by incubating RanGAP1-SUMO1 protein (substrate) immunoprecipitated from *SENP1* ^*−/−*^ T cell lysates with GST-SENP1 WT and its mutants Y270F, Y270E, and C603S (enzyme), respectively, at 37°C for 20 min, followed by immunoblot (top). The densitometric quantification of the ratio of RanGAP1-SUMO1/RanGAP1 is shown below. The input of purified GST-SENP1 proteins was checked by Coomassie staining (bottom). **(F)** *In vivo* SUMO2/3-profiling assay of *SENP1* ^*−/−*^ T cells with rescue expression of YFP-SENP1 WT or its mutants or empty vector. Actin was blotted as loading control. The densitometric quantification of total SUMO2/3 modification (the relative intensity of the control was set as 1) is shown on the right. **(G)** The effect of SENP1 WT and its mutants on the production of IL-2 in T cells. Jurkat E6.1 T cells were transfected with empty vector or YFP-SENP1 WT or its mutants Y270F, Y270E, and C603S and then stimulated with anti-CD3 plus anti-CD28 for 24 h. The concentration of IL-2 in culture supernatants was determined by enzyme-linked immunosorbent assay (ELISA). n.s., not significant. *p < 0.05, **p < 0.01, ***p < 0.001, and ****p < 0.0001 (two-tailed unpaired Student’s *t*-test).

Next, we examined whether SENP1 Y270 phosphorylation could influence its isopeptidase activity. Considering that the presence of endogenous SENP1 may interfere with the effects of ectopic-expressed SENP1, we set to construct a *SENP1* ^*−/−*^ T cell line by CRISPR/Cas9 system. The genome sequencing and western blot results showed that the expression of CRISPR/Cas9-gRNA targeting the first exon of SENP1 induced a thymidine nucleotide insertion in *SENP1* locus to knockout SENP1 gene (Figures 5A,B; Supplementary Figures S3A,B).

Expectedly, *SENP1* ^*−/−*^ T cells displayed much stronger SUMO1 and SUMO2/3 modification (Supplementary Figure S3C). By checking the mRNA expression of SENP2 (another sub-family member of SENP1) in *SENP1* ^*−/−*^ T cells, we found that SENP1 knockout did not affect the transcription of *SENP2* (Supplementary Figure S3D), and western blot showed that the expressions of TCR proximal signaling proteins were not affected in *SENP1* ^*−/−*^ T cells (Supplementary Figure S3E). Therefore, the increased sumoylation in *SENP1* ^*−/−*^ T cells should be the direct result of SENP1 knockout. Global sumoylation reflects the isopeptidase activity of SENP1; hence, we performed an *in vivo* SUMO1-profiling assay in this SENP1 knockout cell line to assess the impact of SENP1 Y270 phosphorylation on its isopeptidase activity (Figure 5C). As expected, SENP1 WT overexpression efficiently reduced the overall sumoylation of cellular proteins, and the capability of SENP1 to remove SUMO1 conjugates from protein substrates was abolished by C603S mutation. Compared with SENP1 WT, the Y270F mutation obviously decreased its desumoylation activity, although this decrease was less than that caused by C603S mutation (Figure 5C), indicating that Y270 tyrosine phosphorylation contributed to the isopeptidase activity of SENP1. Interestingly, the Y270E negative charge mimic mutation did not increase the desumoylation activity of SENP1 but only showed similar isopeptidase activity to SENP1 WT *in vivo* (Figure 5C). A similar pattern of desumoylation activities of SENP1 WT and its mutants is shown in Figure 5D, when analyzing their activities by the ratio of RanGAP1-SUMO1/RanGAP1 in *SENP1* ^*−/−*^ T cells with rescue expression of YFP-SENP1 or its mutants (Figure 5D). Considering the similar activities between SENP1 WT and Y270E mutant, we guessed that in T cells, SENP1 Y270E mutant did not show a stronger activity than SENP1 WT that might result from a certain degree of constitutive Y270 phosphorylation of SENP1 WT. To exclude the possible influence of basal phosphorylation on SENP1, we performed an *in vitro* isopeptidase activity assay, in which *E. coli* recombinant GST fusion proteins of SENP1 WT (which could not be phosphorylated) and its mutants were incubated with immunoprecipitated endogenous total RanGAP1 (including RanGAP1 and RanGAP1-SUMO1) from *SENP1* ^*−/−*^ T cells, respectively (Figure 5E). Basing on the ratio of RanGAP1-SUMO1/RanGAP1, we observed that the desumoylation activity of SENP1 was decreased by both Y270F and C603S mutations, but was increased by Y270E mutation (Figure 5E), indicating Y270 phosphorylation could increase SENP1 isopeptidase activity. Similar results from *in vivo* SUMO2/3-profiling assay also suggested that SENP1 Y270 phosphorylation influenced its isopeptidase activity (Figure 5F). Combining the above *in vivo* and *in vitro* results, we conclude that Y270 phosphorylation is required for the optimal isopeptidase activity of SENP1.

In T cells, SENP1 overexpression inhibits TCR-induced IL-2 production (Wang et al., 2015). Thus, we further examined the effects of Y270 phosphorylation on TCR-induced IL-2 production in Jurkat E6.1 T cells by ELISA (Figure 5G). Consistently, TCR-induced IL-2 production was inhibited by overexpression of SENP1 WT and was increased by C603S mutation, confirming that desumoylation is overall a negative factor in T cell activation. Meanwhile, the IL-2 production was enhanced by Y270F mutation and inhibited by Y270E mutation (Figure 5G), aligned with their desumoylation effects (Figures 5C–F).

### SENP1 Y270 Phosphorylation Influences Its Binding Propensity for Substrates

**FIGURE 6:**
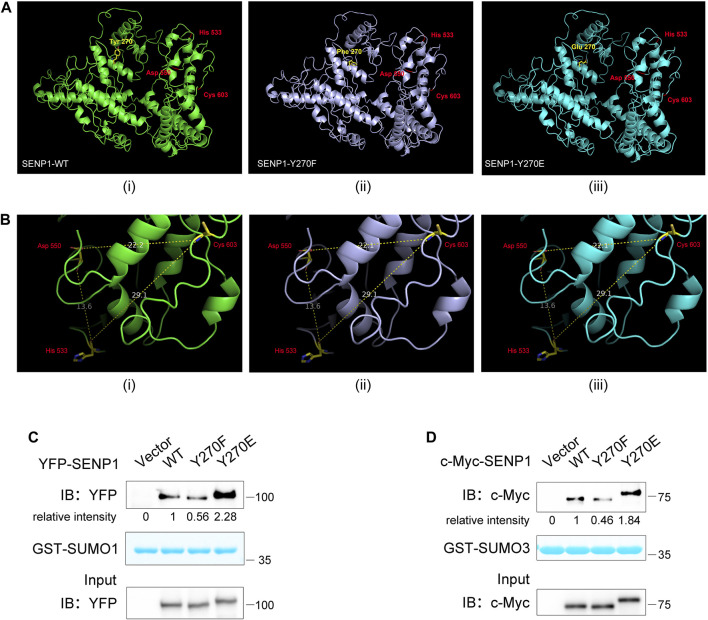
SENP1 Y270 phosphorylation influences its binding propensity for substrates. **(A)** 3D structural models of SENP1 (full length, 1–644 aa, **(i)**, Y270F **(ii)**, and Y270E mutations **(iii)**. **(B)** Distances between key residues in SENP1 WT **(i)**, Y270F **(ii)**, and Y270E structures **(iii)**. The unit of measurement for distance is angstrom (Å). **(C,D)** Analysis of SENP1 WT and its mutants binding to GST-SUMO1 **(C)** and GST-SUMO3 **(D)**. Purified GST-SUMO1 (1–97 aa) **(C)** or GST-SUMO3 (1–92 aa) **(D)** proteins immobilized on glutathione beads were incubated with cell lysate from HEK293T transfected with SENP1 WT or its mutants Y270F and Y270E, followed by western blot analysis (top). The densitometric quantification of bound SENP1 is shown (the relative intensity of SENP1 WT was set as 1). Coomassie staining of GST-SUMO1 or GST-SUMO3 (middle) and immunoblots of WCL of HEK293T transfectants (bottom) are shown.

We next investigated how Y270 phosphorylation regulated SENP1 isopeptidase activity. Firstly, we analyzed whether SENP1 Y270 phosphorylation could directly change its protein conformation, especially the catalytic core domain, to regulate its enzyme activity. The predicted structures of SENP1 WT (1–644 aa), Y270F, and Y270E mutation were obtained, and His533, Asp550, and Cys603 formed the catalytic triad (Figure 6A). We measured the distances between these key residues in these structures and found that neither Y270F nor Y270E mutation brought obvious changes to it (Figure 6B). Since the binding of SUMO1 β-grasp domain (residues 20–92) induces structural changes in the active site of SENP1 and then enhances the enzyme activity of SENP1 (Chen et al., 2014; Guo and Zhou, 2016), the binding of SUMO to SENP1 is hence a prerequisite for SENP1 isopeptidase activity, and we wondered whether Y270 phosphorylation on SENP1 could regulate its isopeptidase activity *via* modulating its binding to SUMO-conjugated substrates. We performed the GST pull-down assay by using GST fused to the N-terminus of mature SUMO1 (1–97 aa) or SUMO3 (1–92 aa). Y270F mutation reduced SENP1 binding to both GST-SUMO1 and -SUMO3. In contrast, Y270E mutation increased its binding to both (Figures 6C,D). These results indicated that Y270 phosphorylation may increase the binding propensity between SENP1 and sumoylated proteins, which can in turn enhance the isopeptidase activity of SENP1. We supposed that allosteric activation of SENP1 by SUMO1 may bring residue 270 closer to SUMO in space, and Y270 phosphorylation may stabilize and enhance the binding between SENP1 and SUMO, which could lead to more efficient desumoylation of the substrates.

## Discussion

**FIGURE 7:**
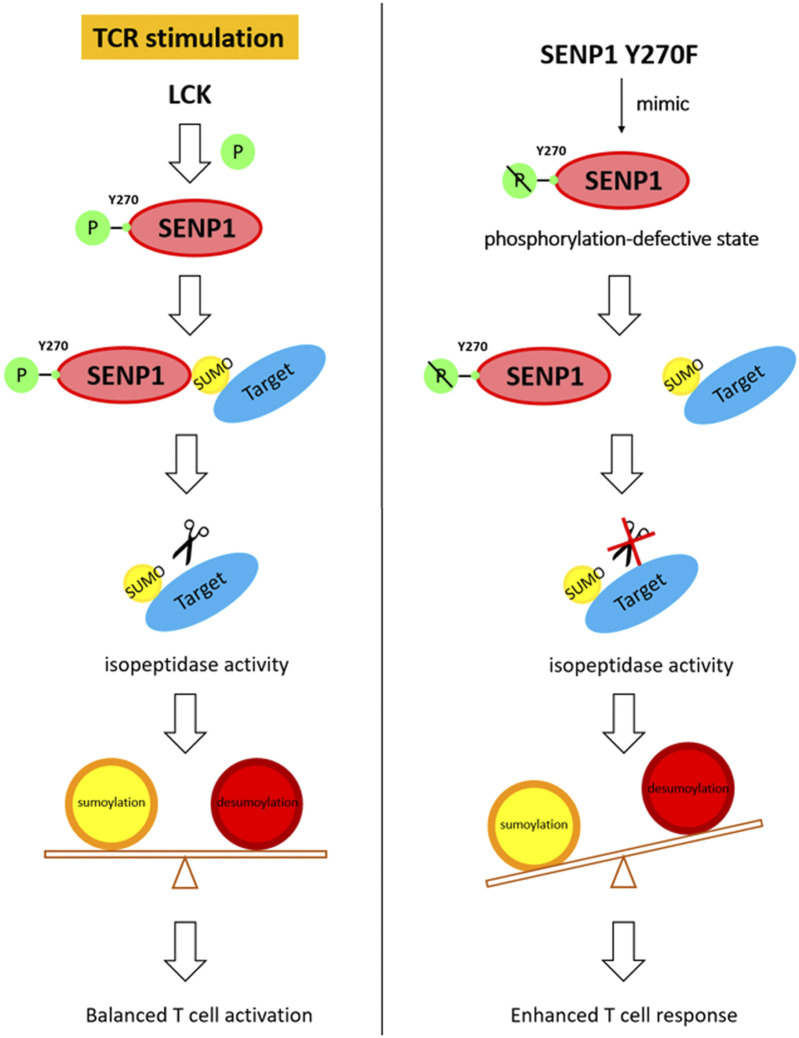
Working model for the regulatory function of SENP1 Y270 phosphorylation.

Sumoylation plays important roles in the regulation of many cellular events. However, how the SUMO system, especially SUMO-specific proteases, is regulated by receptor signaling, such as TCR signaling, remains unclear. In this study, we have found that SENP1 associates with Lck and TCR stimulation induces SENP1 Y270 phosphorylation *via* Lck. This phosphorylation specifically decreases SENP1 endopeptidase activity towards pre-SUMO3 *in vitro* and promotes SENP1 isopeptidase activity by enhancing its binding with both SUMO1- and SUMO2/3-conjugated substrates. Moreover, SENP1 Y270 phosphorylation plays an important regulatory role in T cell activation through reduced overall sumoylation and subsequent inhibition of T cell response. In summary, this study provides the first evidence that TCR signaling could directly phosphorylate SENP1 to modify its enzyme activity and specificity, facilitating fine-tuning of T cell response through balancing the equilibrium between cellular sumoylation and desumoylation (Figure 7).

The N-terminus of SENP1 mainly contributes to SUMO paralogue specificity, and the catalytic core domain of SENP1 is located in its C-terminus (Gong et al., 2000; Chang and Yeh, 2020). SENP1 has been reported to display little specificity in its isopeptidase activity towards different SUMO-conjugated substrates *in vitro* (Shen et al., 2006) and prefers to deconjugate SUMO1- than SUMO2/3-modified substrates in the mouse embryo (Sharma et al., 2013). We have revealed here that in Jurkat T cells, SENP1 could efficiently deconjugate both SUMO1- and SUMO2/3-conjugated substrates, respectively, and SENP1 Y270 phosphorylation enhances its isopeptidase activity towards both *via* increasing its binding propensity to them. Our finding that the non-preference of SENP1 for SUMO1- or SUMO2/3-conjugated substrates may have been caused by the cell types used or overexpression of SENP1 in our experiments. We also observed an interesting result that SENP1 Y270F or Y270E mutants displayed a much clearer phosphorylation effect of SENP1 on RanGAP1-SUMO1 deconjugation *in vitro* than *in vivo* (Figures 5D,E), in line with the findings that SENP1 could deconjugate sumoylated RanGAP1 *in vitro* but not *in vivo* (Gong et al., 2000; Chang and Yeh, 2020), which has been explained as sumoylated RanGAP1 may be protected from SENP1 *in vivo* for being an integral part of the RanBP2 E3 ligase complex (Werner et al., 2012; Chang and Yeh, 2020). Together, our findings have revealed that tyrosine phosphorylation on SENP1 Y270 has a critical role in the regulation of SENP1 isopeptidase activity through increasing its binding propensity with sumoylated proteins.

SENPs also hydrolyzes SUMO precursors in addition to deconjugating sumoylated proteins. Preferring pre-SUMO1 > pre-SUMO2 > pre-SUMO3, SENP1 hydrolyzes SUMO1 precursor in a more efficient way (Shen et al., 2006). The N-terminus helps to achieve this specificity; however, the detailed mechanism is unclear (Xu et al., 2006). We have discovered that Y270 phosphorylation does not affect SENP1 hydrolyzing pre-SUMO1, but, unexpectedly, decreases its hydrolyzing activity towards pre-SUMO3. This finding supplies a regulatory mechanism underlying SENP1 endopeptidase specificity that Y270 phosphorylation of SENP1 could make it focus more on hydrolyzing pre-SUMO1 through weakening its endopeptidase activity towards pre-SUMO3, thereby strengthening its specificity.

Phosphorylation plays important roles in regulation of SENP localization, turn over, and isopeptidase activity. Disturbed blood flow induces SENP2 phosphorylation and promotes its nuclear export in epithelial cells (Heo et al., 2015); mTOR kinase phosphorylates several N-terminal serine/threonine of SENP3 to promote the nucleolar targeting of SENP3 to facilitate ribosome maturation (Raman et al., 2014; Haindl et al., 2008); tumor suppressor protein p19 (Arf) triggers SENP3 to be phosphorylated and then polyubiquitinated and degraded (Kuo et al., 2008). In mitosis, phosphorylation of SENP3 may regulate its enzyme activity and spindle localization (Klein et al., 2009). The C-terminal phosphorylation of yeast SUMO isopeptidase Ulp2 during mitosis may inhibit its isopeptidase activity (Baldwin et al., 2009). Cdk1-mediated Ser/Thr phosphorylation in the N-terminal region of SENP3 in mitotic cells downregulates its deconjugation activity towards topoisomerase TopoIIa through decreasing its interaction with it (Wang et al., 2020; Wei et al., 2018). Different from the above Ser/Thr phosphorylation of SENPs, our study has demonstrated for the first time that in TCR signaling, Lck-mediated tyrosine phosphorylation at SENP1 Y270 regulates the binding propensity between SENP1 and the SUMO-conjugated substrate and then increases SENP1 isopeptidase activity, while blocking the endopeptidase activity towards pre-SUMO3 but not pre-SUMO1 and in turn enhancing SENP1 peptidase specificity. Similar mechanisms for other SENPs in which the enzyme activity and specificity could be modulated by tyrosine phosphorylation in receptor signaling may also exist.

Evolutionary conservation analysis has shown that the Y270 is highly conserved in different species of primates, but not in rodents (Supplementary Figure S1). Guinea pigs (*Cavia porcellus*) has a conserved tyrosine to Y270 in SENP1, but mice and rats do not. Due to the effect of Y270 phosphorylation on SENP1 activity and then T cell activation, it is rational to doubt that Y270 might contribute to different immune characteristics of different species of rodents. It has been revealed that there is greater similarity between humans and guinea pigs than rats or mice in many immunological responses, because guinea pigs and humans are more homologous in some immune-related proteins (Padilla-Carlin et al., 2008). From the perspective of evolutionary conservation, we infer that this phosphorylation-regulated enzyme activity of SENP1 might closely correlate with certain types of immune responses. Further investigations and evidence are needed in the future.

In summary, our findings reveal how SENP1 is regulated by TCR signaling and how Y270 phosphorylation on SENP1 modulates its enzyme activity and specificity and thereby finely tunes the activation of T cells. This study provides a different angle to understand T cell immunology and SENP1 regulation and implies a novel regulatory relationship between receptor signaling and SUMO system (Bailey and O’Hare, 2004, Brownlie and Zamoyska, 2013, Zhao, 2018).

## Data Availability

The original contributions presented in the study are included in the article/Supplementary Material; The mass spectrometry proteomic data presented in the study are deposited in the ProteomeXchange-PRIDE repository, accession number PXD029720 further inquiries can be directed to the corresponding author.

## Supplementary Material

Supplementary Figure S1 Evolutionary conservation analysis of SENP1 Y270 by amino acid sequence alignment of different species.

Supplementary Figure S2 Immunoblotting of purified GST-SUMO3-GFP protein. Purified GST-pSUMO3-GFP protein was incubated with or without GST-SENP1 in endopeptidase activity assay *in vitro*, followed by SDS-PAGE and immunoblotting with anti-GFP (left) and anti-GST (right) antibody.

Supplementary Figure S3 The information of SENP1 knockout cell line. **(A)** The guide sequence targeting the first exon of *SENP1.* **(B)** Flow cytometry analysis of SENP1 knockout in WT and *SENP1* ^*−/−*^ T cells. **(C)** The levels of total SUMO1 and SUMO2/3 modification in WT and *SENP1* ^*−/−*^ T cells examined by immunoblot. Actin was blotted as loading control. **(D)** The mRNA expression levels of *SENP2* in WT and *SENP1* ^*−/−*^ T cells were analyzed by quantitative real-time PCR. **(E)** The expression levels of TCR proximal signaling proteins in WT and *SENP1* ^*−/−*^ T cells. Cells were lysed and then analyzed for the expression level of endogenous proteins Lck, ZAP70, LAT, PLCγ1, PKCθ, and SLP76 by western blot. GAPDH was blotted as loading control.

## References

[B1] Alegre K. O. Reverter D. (2011). Swapping Small Ubiquitin-like Modifier (SUMO) Isoform Specificity of SUMO Proteases SENP6 and SENP7. J. Biol. Chem. 286 (41), 36142–36151. 10.1074/jbc.M111.268847 21878624 PMC3195590

[B2] Bailey D. O'Hare P. (2004). Characterization of the Localization and Proteolytic Activity of the SUMO-specific Protease, SENP1. J. Biol. Chem. 279 (1), 692–703. 10.1074/jbc.M306195200 14563852

[B3] Baldwin M. L. Julius J. A. Tang X. Wang Y. Bachant J. (2009). The Yeast SUMO Isopeptidase Smt4/Ulp2 and the polo Kinase Cdc5 Act in an Opposing Fashion to Regulate Sumoylation in Mitosis and Cohesion at Centromeres. Cell Cycle 8 (20), 3406–3419. 10.4161/cc.8.20.9911 19823017

[B4] Brownlie R. J. Zamoyska R. (2013). T Cell Receptor Signalling Networks: Branched, Diversified and Bounded. Nat. Rev. Immunol. 13 (4), 257–269. 10.1038/nri3403 23524462

[B5] Chang H.-M. Yeh E. T. H. (2020). SUMO: From Bench to Bedside. Physiol. Rev. 100 (4), 1599–1619. 10.1152/physrev.00025.2019 32666886 PMC7717128

[B6] Chen C.-H. Namanja A. T. Chen Y. (2014). Conformational Flexibility and Changes Underlying Activation of the SUMO-specific Protease SENP1 by Remote Substrate Binding. Nat. Commun. 5, 4968. 10.1038/ncomms5968 25263960 PMC4285349

[B7] Creton S. Jentsch S. (2010). SnapShot: The SUMO System. Cell 143 (5), 848. 10.1016/j.cell.2010.11.026 21111242

[B8] Ding X. Wang A. Ma X. Demarque M. Jin W. Xin H. (2016). Protein SUMOylation Is Required for Regulatory T Cell Expansion and Function. Cel Rep. 16 (4), 1055–1066. 10.1016/j.celrep.2016.06.056 27425617

[B9] Flotho A. Melchior F. (2013). Sumoylation: A Regulatory Protein Modification in Health and Disease. Annu. Rev. Biochem. 82, 357–385. 10.1146/annurev-biochem-061909-093311 23746258

[B10] Geiss-Friedlander R. Melchior F. (2007). Concepts in Sumoylation: A Decade on. Nat. Rev. Mol. Cel Biol 8 (12), 947–956. 10.1038/nrm2293 18000527

[B11] Gong L. Millas S. Maul G. G. Yeh E. T. H. (2000). Differential Regulation of Sentrinized Proteins by a Novel Sentrin-specific Protease. J. Biol. Chem. 275 (5), 3355–3359. 10.1074/jbc.275.5.3355 10652325

[B12] Gong L. Yeh E. T. H. (2006). Characterization of a Family of Nucleolar SUMO-specific Proteases with Preference for SUMO-2 or SUMO-3. J. Biol. Chem. 281 (23), 15869–15877. 10.1074/jbc.M511658200 16608850

[B13] Guerra de Souza A. C. Prediger R. D. Cimarosti H. (2016). SUMO-Regulated Mitochondrial Function in Parkinson's Disease. J. Neurochem. 137 (5), 673–686. 10.1111/jnc.13599 26932327

[B14] Guo J. Zhou H.-X. (2016). Allosteric Activation of SENP1 by SUMO1 β-grasp Domain Involves a Dock-And-Coalesce Mechanism. eLife 5, e18249. 10.7554/eLife.18249 27576863 PMC5030089

[B15] Haindl M. Harasim T. Eick D. Muller S. (2008). The Nucleolar SUMO-specific Protease SENP3 Reverses SUMO Modification of Nucleophosmin and Is Required for rRNA Processing. EMBO Rep. 9 (3), 273–279. 10.1038/embor.2008.3 18259216 PMC2267381

[B16] He Y. Yang Z. Zhao C.-s. Xiao Z. Gong Y. Li Y.-Y. (2021). T-cell Receptor (TCR) Signaling Promotes the Assembly of RanBP2/RanGAP1-SUMO1/Ubc9 Nuclear Pore Subcomplex via PKC-θ-Mediated Phosphorylation of RanGAP1. eLife 10, e67123. 10.7554/eLife.67123 34110283 PMC8225385

[B17] Heo K.-S. Le N.-T. Cushman H. J. Giancursio C. J. Chang E. Woo C.-H. (2015). Disturbed Flow-Activated p90RSK Kinase Accelerates Atherosclerosis by Inhibiting SENP2 Function. J. Clin. Invest. 125 (3), 1299–1310. 10.1172/JCI76453 25689261 PMC4362265

[B18] Hickey C. M. Wilson N. R. Hochstrasser M. (2012). Function and Regulation of SUMO Proteases. Nat. Rev. Mol. Cel Biol 13 (12), 755–766. 10.1038/nrm3478 PMC3668692 23175280

[B19] Ittisoponpisan S. Islam S. A. Khanna T. Alhuzimi E. David A. Sternberg M. J. E. (2019). Can Predicted Protein 3D Structures Provide Reliable Insights into whether Missense Variants Are Disease Associated?. J. Mol. Biol. 431 (11), 2197–2212. 10.1016/j.jmb.2019.04.009 30995449 PMC6544567

[B20] Johnson E. S. (2004). Protein Modification by SUMO. Annu. Rev. Biochem. 73, 355–382. 10.1146/annurev.biochem.73.011303.074118 15189146

[B21] Karhausen J. Ulloa L. Yang W. (2021). SUMOylation Connects Cell Stress Responses and Inflammatory Control: Lessons from the Gut as a Model Organ. Front. Immunol. 12, 646633. 10.3389/fimmu.2021.646633 33679811 PMC7933481

[B22] Kim J. H. Baek S. H. (2009). Emerging Roles of Desumoylating Enzymes. Biochim. Biophys. Acta (Bba) - Mol. Basis Dis. 1792 (3), 155–162. 10.1016/j.bbadis.2008.12.008 19162180

[B23] Klein U. R. Haindl M. Nigg E. A. Muller S. (2009). RanBP2 and SENP3 Function in a Mitotic SUMO2/3 Conjugation-Deconjugation Cycle on Borealin. Mol. Biol. Cel 20 (1), 410–418. 10.1091/mbc.e08-05-0511 PMC2613119 18946085

[B24] Kunz K. Piller T. Müller S. (2018). SUMO-specific Proteases and Isopeptidases of the SENP Family at a Glance. J. Cel Sci. 131 (6), jcs211904. 10.1242/jcs.211904 29559551

[B25] Kuo M.-L. den Besten W. Thomas M. C. Sherr C. J. (2008). Arf-induced Turnover of the Nucleolar Nucleophosmin-Associated SUMO-2/3 Protease Senp3. Cell Cycle 7 (21), 3378–3387. 10.4161/cc.7.21.6930 18948745

[B26] Lima C. D. Reverter D. (2008). Structure of the Human SENP7 Catalytic Domain and Poly-SUMO Deconjugation Activities for SENP6 and SENP7. J. Biol. Chem. 283 (46), 32045–32055. 10.1074/jbc.M805655200 18799455 PMC2581585

[B27] Liu B. Shuai K. (2009). Summon SUMO to Wrestle with Inflammation. Mol. Cel 35 (6), 731–732. 10.1016/j.molcel.2009.09.002 19782020

[B28] Liu H. Schneider H. Recino A. Richardson C. Goldberg M. W. Rudd C. E. (2015). The Immune Adaptor SLP-76 Binds to SUMO-RANGAP1 at Nuclear Pore Complex Filaments to Regulate Nuclear Import of Transcription Factors in T Cells. Mol. Cel 59 (5), 840–849. 10.1016/j.molcel.2015.07.015 PMC4576164 26321253

[B29] Madu I. G. Chen Y. (2012). Assays for Investigating deSUMOylation Enzymes. Curr. Protoc. Mol. Biol. 99, t10–t30. 10.1002/0471142727.mb1030s99 PMC3711413 22870856

[B30] Madu I. G. Namanja A. T. Su Y. Wong S. Li Y.-J. Chen Y. (2013). Identification and Characterization of a New Chemotype of Noncovalent SENP Inhibitors. ACS Chem. Biol. 8 (7), 1435–1441. 10.1021/cb400177q 23614497 PMC3840147

[B31] Mendler L. Braun T. Müller S. (2016). The Ubiquitin-like SUMO System and Heart Function. Circ. Res. 118 (1), 132–144. 10.1161/CIRCRESAHA.115.307730 26837744

[B32] Mikolajczyk J. Drag M. Békés M. Cao J. T. Ronai Z. e. Salvesen G. S. (2007). Small Ubiquitin-Related Modifier (SUMO)-specific Proteases. J. Biol. Chem. 282 (36), 26217–26224. 10.1074/jbc.M702444200 17591783

[B33] Nayak A. Müller S. (2014). SUMO-specific Proteases/isopeptidases: SENPs and beyond. Genome Biol. 15 (7), 422. 10.1186/s13059-014-0422-2 25315341 PMC4281951

[B34] Owerbach D. McKay E. M. Yeh E. T. H. Gabbay K. H. Bohren K. M. (2005). A Proline-90 Residue Unique to SUMO-4 Prevents Maturation and Sumoylation. Biochem. Biophysical Res. Commun. 337 (2), 517–520. 10.1016/j.bbrc.2005.09.090 16198310

[B35] Padilla-Carlin D. J. McMurray D. N. Hickey A. J. (2008). The guinea Pig as a Model of Infectious Diseases. Comp. Med. 58 (4), 324–340. 18724774 PMC2706043

[B36] Raman N. Nayak A. Muller S. (2014). MTOR Signaling Regulates Nucleolar Targeting of the SUMO-specific Isopeptidase SENP3. Mol. Cel. Biol. 34 (24), 4474–4484. 10.1128/MCB.00801-14 PMC4248736 25288641

[B37] Reverter D. Lima C. D. (2004). A Basis for SUMO Protease Specificity provided by Analysis of Human Senp2 and a Senp2-SUMO Complex. Structure 12 (8), 1519–1531. 10.1016/j.str.2004.05.023 15296745

[B38] Reverter D. Lima C. D. (2006). Structural Basis for SENP2 Protease Interactions with SUMO Precursors and Conjugated Substrates. Nat. Struct. Mol. Biol. 13 (12), 1060–1068. 10.1038/nsmb1168 17099700

[B39] Roy A. Kucukural A. Zhang Y. (2010). I-TASSER: A Unified Platform for Automated Protein Structure and Function Prediction. Nat. Protoc. 5 (4), 725–738. 10.1038/nprot.2010.5 20360767 PMC2849174

[B40] Sanjana N. E. Shalem O. Zhang F. (2014). Improved Vectors and Genome-wide Libraries for CRISPR Screening. Nat. Methods 11 (8), 783–784. 10.1038/nmeth.3047 25075903 PMC4486245

[B41] Seeler J.-S. Dejean A. (2017). SUMO and the Robustness of Cancer. Nat. Rev. Cancer 17 (3), 184–197. 10.1038/nrc.2016.143 28134258

[B42] Shah N. H. Löbel M. Weiss A. Kuriyan J. (2018). Fine-tuning of Substrate Preferences of the Src-Family Kinase Lck Revealed through a High-Throughput Specificity Screen. eLife 7, e35190. 10.7554/eLife.35190 29547119 PMC5889215

[B43] Shah N. H. Wang Q. Yan Q. Karandur D. Kadlecek T. A. Fallahee I. R. (2016). An Electrostatic Selection Mechanism Controls Sequential Kinase Signaling Downstream of the T Cell Receptor. eLife 5, e20105. 10.7554/eLife.20105 27700984 PMC5089863

[B44] Shalem O. Sanjana N. E. Hartenian E. Shi X. Scott D. A. Mikkelsen T. S. (2014). Genome-scale CRISPR-Cas9 Knockout Screening in Human Cells. Science 343 (6166), 84–87. 10.1126/science.1247005 24336571 PMC4089965

[B45] Sharma P. Yamada S. Lualdi M. Dasso M. Kuehn M. R. (2013). Senp1 Is Essential for Desumoylating Sumo1-Modified Proteins but Dispensable for Sumo2 and Sumo3 Deconjugation in the Mouse Embryo. Cel Rep. 3 (5), 1640–1650. 10.1016/j.celrep.2013.04.016 PMC3775507 23684609

[B46] Shen L. N. Dong C. Liu H. Naismith J. H. Hay R. T. (2006). The Structure of SENP1-SUMO-2 Complex Suggests a Structural Basis for Discrimination between SUMO Paralogues during Processing. Biochem. J. 397 (2), 279–288. 10.1042/BJ20052030 16553580 PMC1513277

[B47] Shen L. N. Geoffroy M.-C. Jaffray E. G. Hay R. T. (2009). Characterization of SENP7, a SUMO-2/3-specific Isopeptidase. Biochem. J. 421 (2), 223–230. 10.1042/BJ20090246 19392659

[B48] Wang Q.-L. Liang J.-Q. Gong B.-N. Xie J.-J. Yi Y.-T. Lan X. (2019). T Cell Receptor (TCR)-Induced PLC-Γ1 Sumoylation via PIASxβ and PIAS3 SUMO E3 Ligases Regulates the Microcluster Assembly and Physiological Function of PLC-Γ1. Front. Immunol. 10, 314. 10.3389/fimmu.2019.00314 30873169 PMC6403162

[B49] Wang X.-D. Gong Y. Chen Z.-L. Gong B.-N. Xie J.-J. Zhong C.-Q. (2015). TCR-induced Sumoylation of the Kinase PKC-θ Controls T Cell Synapse Organization and T Cell Activation. Nat. Immunol. 16 (11), 1195–1203. 10.1038/ni.3259 26390157

[B50] Wang Y. Tian J. Huang C. Ma J. Hu G. Chen Y. (2020). P53 Suppresses SENP3 Phosphorylation to Mediate G2 Checkpoint. Cell Discov 6, 21. 10.1038/s41421-020-0154-2 32351703 PMC7171148

[B51] Wei B. Huang C. Liu B. Wang Y. Xia N. Fan Q. (2018). Mitotic Phosphorylation of SENP3 Regulates DeSUMOylation of Chromosome-Associated Proteins and Chromosome Stability. Cancer Res. 78 (9), 2171–2178. 10.1158/0008-5472.CAN-17-2288 29438989

[B52] Werner A. Flotho A. Melchior F. (2012). The RanBP2/RanGAP1*SUMO1/Ubc9 Complex Is a Multisubunit SUMO E3 Ligase. Mol. Cel 46 (3), 287–298. 10.1016/j.molcel.2012.02.017 22464730

[B53] Xiong Y. Yi Y. Wang Y. Yang N. Rudd C. E. Liu H. (2019). Ubc9 Interacts with and SUMOylates the TCR Adaptor SLP-76 for NFAT Transcription in T Cells. J. Immunol. 203 (11), 3023–3036. 10.4049/jimmunol.1900556 31666306

[B54] Xu Z. Au S. W. N. (2005). Mapping Residues of SUMO Precursors Essential in Differential Maturation by SUMO-specific Protease, SENP1. Biochem. J. 386 (Pt 2), 325–330. 10.1042/BJ20041210 15487983 PMC1134797

[B55] Xu Z. Chau S. F. Lam K. H. Chan H. Y. Ng T. B. Au S. W. N. (2006). Crystal Structure of the SENP1 Mutant C603S-SUMO Complex Reveals the Hydrolytic Mechanism of SUMO-specific Protease. Biochem. J. 398 (3), 345–352. 10.1042/BJ20060526 16712526 PMC1559472

[B56] Yang J. Yan R. Roy A. Xu D. Poisson J. Zhang Y. (2015). The I-TASSER Suite: Protein Structure and Function Prediction. Nat. Methods 12 (1), 7–8. 10.1038/nmeth.3213 25549265 PMC4428668

[B57] Yang J. Zhang Y. (2015). I-TASSER Server: New Development for Protein Structure and Function Predictions. Nucleic Acids Res. 43 (W1), W174–W181. 10.1093/nar/gkv342 25883148 PMC4489253

[B58] Zhao X. (2018). SUMO-mediated Regulation of Nuclear Functions and Signaling Processes. Mol. Cel 71 (3), 409–418. 10.1016/j.molcel.2018.07.027 PMC6095470 30075142

