# Supplementary material for: TCR-Induced Tyrosine Phosphorylation at Tyr270 of SUMO Protease SENP1 by Lck Modulates SENP1 Enzyme Activity and Specificity
Source: Front Cell Dev Biol. 2022 Feb 2;9:789348. doi: 10.3389/fcell.2021.789348 (PMC8847397; doi:10.3389/fcell.2021.789348)
Supplement: Supplementary file 2 [file Table1.docx]

**[Supplementary Table 1](https://www.frontiersin.org/articles/10.3389/fcell.2020.00469/full#TS1)**

| Primer/sgRNA designation | Sequence |
| --- | --- |
| SENP1 | F: ATGGATGATATTGCTGATAG  R: CAAGAGTTTTCGGTGGAGGATC |
| Lck | F: ATGGGCTGTGGCTGCAGCTCACAC  R: AGGCTGAGGCTGGTACTGGCCCTCT |
| ZAP70 | F: ATGCCAGACCCCGCGGCGCACCTGC  R: GGCACAGGCAGCCTCAGCCTTCTGT |
| SUMO1 1-97aa | F: ATGTCTGACCAGGAGGCAAAACC  R: ACCCCCCGTTTGTTCC |
| SUMO3 1-92 aa | F: ATGTCCGAGGAGAAGCCCAAG  R: ACCTCCCGTCTGCTGC |
| SENP1 Y119F | F: GAAACAGCCGAAGTCTTTTCCTCGAAACCCGAAAGAC  R: GTCTTTCGGGTTTCGAGGAAAAGACTTCGGCTGTTTC |
| SENP1 Y194F | F: GAAGAAAGAGAGATTTTCAGACAGCTGCTACAG  R: CTGTAGCAGCTGTCTGAAAATCTCTCTTTCTTC |
| SENP1 Y270F | F: GCTGTCCCACAGTGTATTCTCCCTATCTTCTTATACC  R: GGTATAAGAAGATAGGGAGAATACACTGTGGGACAGC |
| SENP1 Y270E | F: CAGCTGTCCCACAGTGTAGAGTCCCTATCTTCTTATAC  R: GTATAAGAAGATAGGGACTCTACACTGTGGGACAGCTG |
| SENP1 Y349F | F: GAATTAACTAGTGTTTTCGATTCTCGAGCACGAG  R: CTCGTGCTCGAGAATCGAAAACACTAGTTAATTC |
| SENP1 C603S | F: GATGAATGGAAGTGACGCCGGGATGTTTGCCTGC  R: GCAGGCAAACATCCCGGCGTCACTTCCATTCATC |
| Lck K273R | F: GGTGGCGGTGAGAAGCCTGAAGCAG  R: CTGCTTCAGGCTTCTCACCGCCACC |
| SENP1 sgRNA | GCTTTCGCTTTCTGACCAGC |
| SENP1 Genomic primer | F: GAACTAATCCAGACTGTCTCC  R: TTAAAGGAAGAAAATTGCTCC |
| Human SENP2 qPCR primer | F: CTCAGGAACAGGCTGTAACA  R: CAGGACAGACAGAGTTTCCA |
| Human GAPDH qPCR primer | F:CATTGCCCTCAACGACCACTTTGT  R:TCTCTCTCTTCCTCTTGTGCTCTTGC |
