# Supplementary figures and images for: TCR-Induced Tyrosine Phosphorylation at Tyr270 of SUMO Protease SENP1 by Lck Modulates SENP1 Enzyme Activity and Specificity
Source: Front Cell Dev Biol. 2022 Feb 2;9:789348. doi: 10.3389/fcell.2021.789348 (PMC8847397; doi:10.3389/fcell.2021.789348)

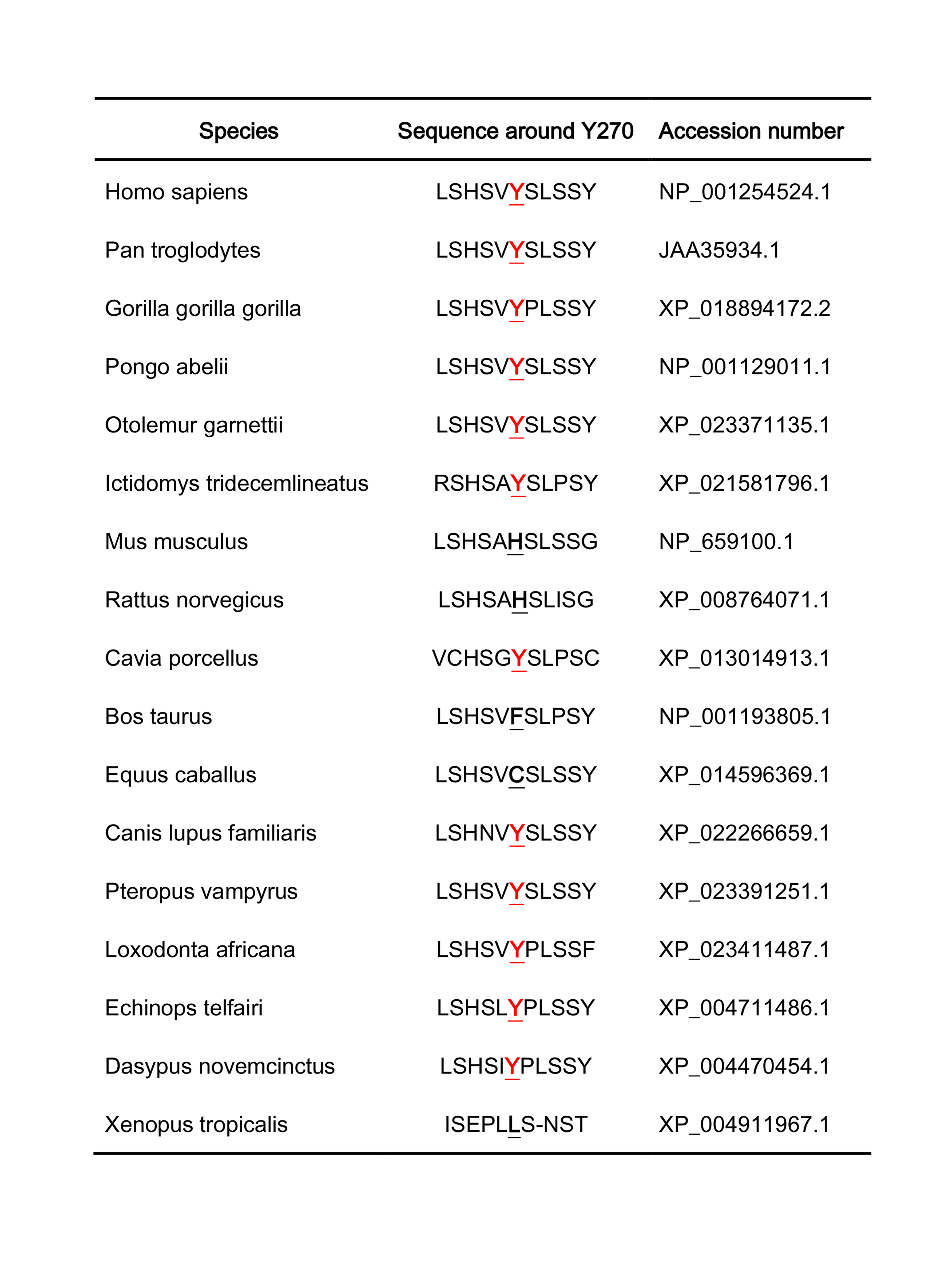

Supplement: Supplementary file 3 [file Image1.JPEG]

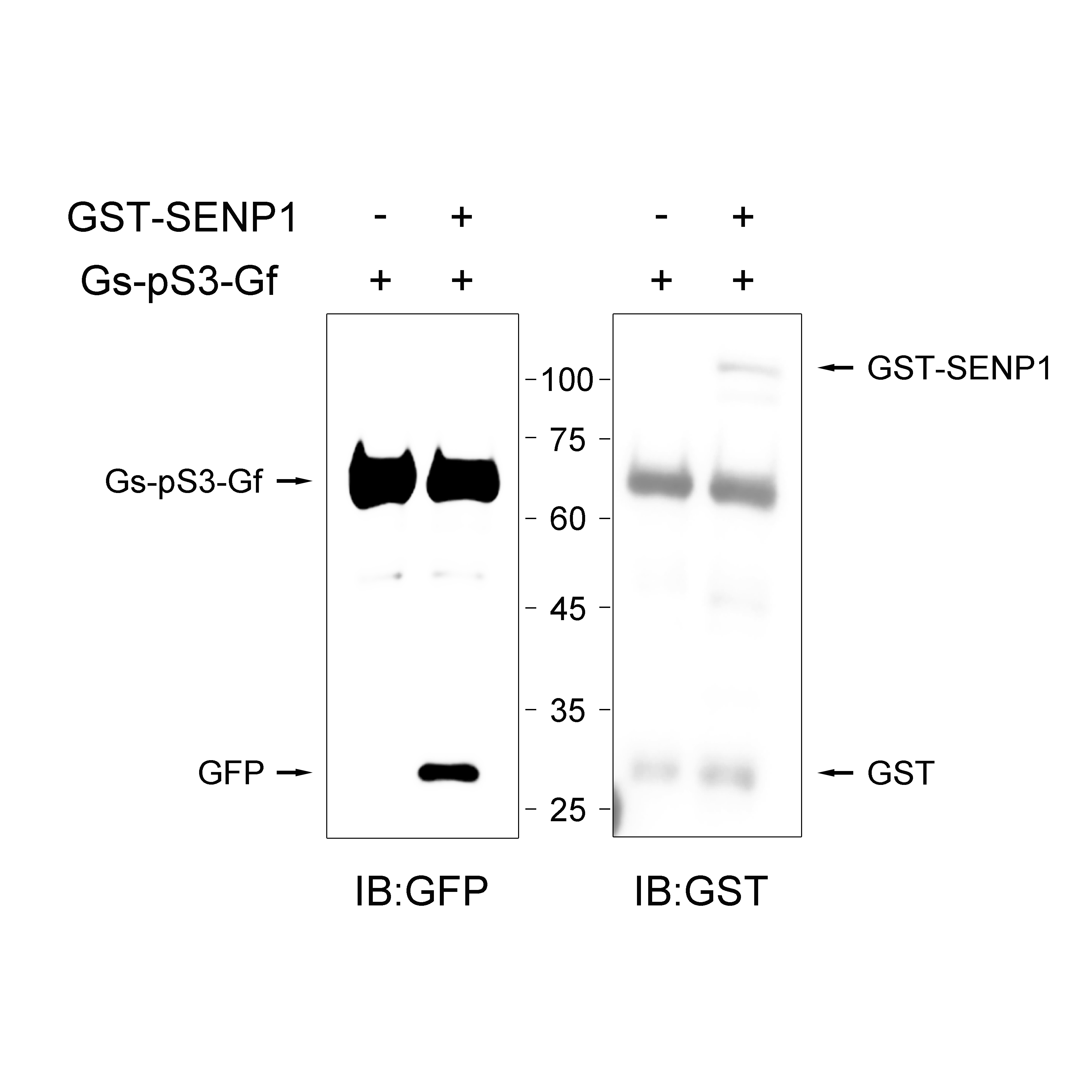

Supplement: Supplementary file 4 [file Image2.JPEG]
